# Conference report from the 2015 OECI Oncology Days, Portugal, 22–24 June—tumour heterogeneity and next generation sequencing: morphology and technology

**DOI:** 10.3332/ecancer.2015.565

**Published:** 2015-08-19

**Authors:** Linda Cairns

**Affiliations:** Science Editor, ecancermedicalscience, European Institute of Oncology, Via Ripamonti 435, Milan 20141, Italy; * on behalf of the participants

**Keywords:** intertumoural heterogeneity, intratumoural heterogeneity, predictive biomarkers, cancer diagnosis

## Abstract

Tumour heterogeneity was the topic of the ‘Oncology Days’ series held at the 2015 OECI conference in which experts within the field provided an update on tumour heterogeneity and its relevance in the clinical setting.

Here we present a summary of the presentations from the two major sessions of the meeting: clonal heterogeneity and phenotypic heterogeneity.

## Introduction

Tumour heterogeneity describes differences between tumours of apparently the same type but in different host backgrounds (intertumoural heterogeneity) and differences between cancer cells within the same tumour (intratumoural heterogeneity). In both cases, this heterogeneity can lead to different responses to therapy.

Evidence for intratumoural heterogeneity was described back in the 1980s [1, 2, 3], but now clinicians realise that characterisation of heterogeneity will be important to better understand the development and progression of tumours and the best therapeutic options.

Genetic and epigenetic differences between cancer cells within a tumour might explain why some tumour cells are more aggressive and difficult to eradicate in patients treated with chemotherapy. In fact intratumour heterogeneity may also explain why some patients who initially respond well to a particular drug often relapse and develop new tumours which do not respond to the therapy.

## Clonal heterogeneity

## Can heterogeneity affect predictive biomarker diagnosis? *Marco Gerlinger*

Most tumours initiate from a single cell which has acquired driver mutations necessary for malignant transformation. However, additional mutations can be acquired during the neoplastic process leading to genetic diversity within the tumour population i.e. heterogeneity. This genetic diversity is the substrate for adaptation to changing environments and for the development of drug resistance through Darwinian evolution. Recent work from our group and others has shown that genetic intratumour heterogeneity and branched evolution is common in many solid tumour types. These are major hurdles for the development of better treatment strategies and prognostic and predictive biomarkers for personalised cancer medicine approaches.

We are interested in the impact of genetic and non-genetic intratumour heterogeneity on disease progression and how systemic therapies reshape cancer genomic landscapes. This should reveal the principles governing cancer evolution and should eventually translate into novel therapeutic strategies which can influence evolutionary trajectories for e.g. to prevent or delay the development of drug resistance. The development of biomarkers which are applicable to heterogeneous cancers, for e.g. through quantification of circulating tumour DNA, is a further priority of the group.

Heterogeneity is a problem for treatment as these different clones are usually spatially separated within the tumour and leads to drug resistance.

In order to avoid giving patients drugs that have little likelihood of working, we need to think about tumours in a different way. Thinking of the tumour as a heterogeneic unit may improve biomarker accuracy.

## Why study tumour heterogeneity from a practical point of view in diagnostic and clinical research? *Giorgio Stanta*

There are different types of tumour heterogeneity: macroscopic i.e. clinical and ethnic heterogeneity which is very well recognised and widely reported. On a microscopic level, pathologists now recognise different histologic patterns within a single tumour with specific morphological characteristics. These characteristics lead to intertumour heterogeneity, but the same features may also lead to intratumour differences, such as different histological and cytological patterns in the same tumour with different degrees of differentiation with prognostic significance. What is more, it is known that in the same tumour there are different functional areas such as the centre or the infiltrative border of the tumour, also with different expression patterns. However, today tumour heterogeneity is mostly related to molecular differences at the inter- and intratumour level. It is the latter, intratumour heterogeneity (ITH) that can explain acquired resistance to cytotoxic chemotherapy or to new targeted drugs, and it occurs so often in tumours that in most of the cases it is the cause of treatment failure.

At the moment, we do not have any precise indication on how to standardise this kind of heterogeneity, but we know that this ITH can be studied by using specific methodologies like single-cell sequencing or next generation sequencing (NGS) analysis from different tumour areas or *in-situ* methods such as *in situ* hybridisation (ISH), or immunohistochemistry (IHC), or fluorescence *in situ* hybridisation (FISH). Also molecular heterogeneity can be homotypic or heterotypic, and in the latter case it important to recognise the histological components of the microenvironment. Hopefully, by comparing what is common and what is different in the various tumour types will lead to specific characteristics which are more frequent at the level of pathology.

We have to consider two different situations in which heterogeneity may influence prognosis and patient treatment. The first one is related to the primary tumour acquired after surgery which can give us information about ITH. We know that sometimes the killer clone is a minor one with less aggressive behaviour. For this reason, a qualified analysis of primary tumour subclones would allow a rational treatment to avoid recurrences. The second one is related to patients with advanced cancer or recurrence in which liquid biopsies can be extremely important to try to understand which are the best targets for therapy. ITH indeed is evident in cancer spreading; metastatic lesions are different at different secondary sites resulting in intermetastatic heterogeneity, but it can also be heterogeneous within a single metastasis and it is called intrametastatic heterogeneity. The comparison of molecular characteristics between primary tumours and their corresponding metastatic sites, even synchronous local lymph node metastases, can help to understand heterogeneity and also at the same time the biology of the metastatic process.

Variation between primary tumours and metastasis could probably be explained by many factors ranging from a clonal selection in the metastatisation process, to the phenotypic plasticity related to the new microenvironment which can interact with the tumour cells, in a process of ‘inverted’ mesenchymal/epithelial transition. Metastases are supposed to derive from a single cell or subclone, but recently also polyclonal seeding from one metastasis to another was detected.

A big improvement in the knowledge of cancer spreading comes from so-called ‘liquid biopsies’. Circulating tumour cells (CTC) and circulating free DNA (cfDNA) are huge contributors in the biological and clinical understanding of cancer recurrences and acquired mechanisms of resistance. In this process, ITH has a central position because low frequency clones can be resistant to therapy and could be the basis of therapy failure. These clones can be detected in blood during the follow-up of treatment outcomes, and the therapy itself represents the selection pressure factor for clonal evolution. Secondary acquired resistance to treatment is related to defined genetic or phenotypic heterogeneous mechanisms that are similar in different tumour types. Today the limits of liquid biopsies are still uncertain and connected with tumour ITH. Cells and DNA in blood can come from different and heterogeneous metastatic sites; sensitivity, and the reproducibility in detecting tumour clonal range still has to be proved.

## How can tumour heterogeneity modify cancer diagnostics? *Manfred Dietel*

**Figure 1.**
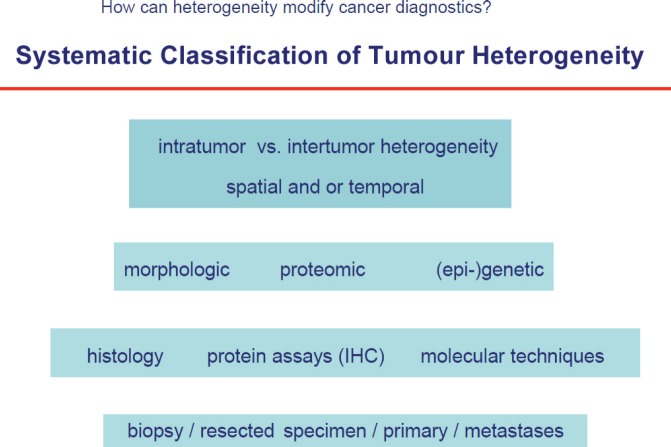
Systemic classification of tumour heterogeneity.

Human tumours are lesions which almost always develop over several years, e.g. for breast cancer it was shown that it needs approximately ten years from the first malignant cell until the lesion is clinically detectable. During this period roughly 109–1012 cell divisions take place which can produce a plethora of genetic alterations resulting in a high number of different cell clones. This is one major mechanism underlying not only intratumour heterogeneity, but also differences among different tumours that originate in the same organ (intertumour heterogeneity, Figure 1. Herein, heterogeneity not only applies to genetic changes, but is present also on other levels of molecular or phenotypic properties, such as epigenetic or proteomic tumour profiles. Moreover, tumour heterogeneity is influenced by temporal and spatial (microenvironmental) factors and is therefore present on all functional levels of tumour cells [4].

The techniques to study tumour heterogeneity are multifaceted and include basic histology, IHC, FISH as well as NGS to detect diverse genetic alterations (amplicon, whole exome or whole genome) or epigenome-wide association studies (EWAS), integration of whole proteome or sub-proteome profiles—mass spectrometry, protein arrays, reversed protein arrays (beads) etc. and functional assays. Detection, quantification, and characterisation of methylome patterns and long non-coding RNAs (potential utility of 450K arrays as broad tumour classification tool based on methylation levels) will complete the approach. These methods will be applied to tissue but also to tumour derived nucleic acids (ctDNA) or proteins in plasma, urine, sputum, and spinal fluid or in CTCs. These approaches are summarised under the term liquid biopsies.

However, because of technical and financial limitations it will not be possible to apply all the techniques mentioned above in the foreseeable future in routine diagnostics. In the opinion of the author, three novel approaches exist which will have to be introduced in daily routine procedures as soon as possible.

1. Computer-based image analyses to quantify immunohistochemical stains. Because of the fact that the human eye is neither an ideal ‘instrument’ to quantify colour intensity nor to estimate percentage positivity of structures, e.g. nuclei or membrane staining, the inter- and intra-observer variability in the evaluation of therapy relevant structures is high. Digital scanning of slides together with virtual microscopy and intelligent algorithms, e.g. object-based image analyses [5], will become extremely helpful in overcoming this problem and will contribute to a higher rate of reproducibility of quantitative IHC evaluation, e.g. for HER-2 or anaplastic lymphoma kinase (ALK).
2. NGS is becoming a routinely applied method for genetic profiling of malignant tumours in diagnostic molecular pathology. Currently, this may be restricted to so called druggable mutations [6], i.e. mutations which can be targeted by particular drugs but will be extended to more comprehensive sequencing approaches in the coming years (‘discovery diagnostics’). In particular, for rare tumours molecular profiling will open the door for many patients to receive efficient targeted drugs in an off-label status.
3. Introduction of liquid biopsies for follow-up or the continuous ‘real time’ analysis of heterogeneity that develops under therapy. It is generally known that because of mutational instability of tumour cells and/or drug-induced selection, drug resistance almost always develops under therapy. This process is independent of whether conventional chemotherapy or novel targeted drugs are applied. The ability to monitor the cellular changes and/or clonal evolution of the disease is becoming a novel challenge and may fundamentally influence the course of drug applications.

Based on the new possibilities the establishment of molecular staging, follow-up of disease, tracing resistance, predicting therapy will become feasible.

## Conclusions

In the daily clinical work, tumour heterogeneity may influence cancer diagnostics by

- leading to an incomplete histological diagnosis,
- an incomplete immunoprofiling of malignant tumours which could have consequences for treatment decisions, in particular drug selection, and
- by over—or underestimation of genetic alterations that in turn could have consequences for (targeted) treatment

The most relevant question is how to cope with tumour heterogeneity in clinical practice:

- In histological diagnostics as many specimen regions as economically justifiable (several biopsies of several locus of a resection specimen) should be evaluated.
- As many loci as economically justifiable should be used for immunoprofiling and should be complemented by,
- image analysis to reach a higher degree of reproducibility in tissue feature quantification.
- Genetic alterations should be investigated in at least several (e.g. n = 5) loci.
- Molecular profiling by NGS and in future other techniques should become a routine procedure in the diagnostic process of malignant diseases.

## Colorectal cancer molecular subtypes: from patients to preclinical models and back, the problem of heterogeneity *Enzo Medico*

Colorectal cancer (CRC) is a heterogeneous disease, displaying variable molecular marks and clinical behaviour. Recent studies have used gene expression profiles to identify colorectal cancer transcriptional subtypes endowed with distinctive molecular, biological, and clinical features. Among the subtypes, the stem/serrated/mesenchymal (SSM) one is characterised by expression of stem/mesenchymal cell transcripts and poor prognosis. Although it has been suggested that this subtype is comprised of cancer cells that have undergone a switch from an epithelial to a mesenchymal/stem cell–like state, we noted that genes upregulated in this subtype are also prominently expressed by stromal cells. This led us to hypothesise that the SSM transcripts could derive from the tumour microenvironment rather than being intrinsic to cancer cells. To test this hypothesis, we analysed RNA sequencing data from patient-derived xenografts (PDXs) of CRC, where human cancer cells are supported by mouse stroma. By using the mouse read fraction as a proxy for the stromal contribution to the expression of a gene, we found that stromal (mouse) transcripts were much more abundant than CRC epithelial (human) transcripts for the vast majority of the SSM signature genes, indicating that tumour-associated stromal cells are the main driver of the SSM class. Considering only genes not detected as human transcripts in PDXs, we built three expression signatures specifically reporting the abundance of cancer-associated fibroblasts (CAFs), leucocytes, or endothelial cells. In human CRC samples, all stromal signatures were strongly associated with the SSM subtype. A high CAF signature was associated with poor prognosis in untreated CRC patients, while in rectal cancer high stromal signatures jointly predicted radioresistance. Our results show that the distinctive transcriptional and clinical features of the SSM subtype can be ascribed to its particularly abundant stroma. The ambiguity between stromal cells and mesenchymal tumour cells can be addressed through RNA sequencing of xenograft models, where the expression characteristics of the human tumour cells can be distinguished from those of the mouse microenvironment *in silico*.

## Phenotypic heterogeneity

## Molecular heterogeneity of prostate cancer *Manuel R. Teixeira*

Prostate carcinoma (PCa) is a heterogeneous disease at several levels, of which some were addressed in this presentation, namely heterogeneity in hereditary predisposition, intertumour heterogeneity of gene fusions, intra-tumour heterogeneity of gene fusions, genetic markers of prognosis, and molecular subtyping and targeted therapy.

Regarding heterogeneity in inherited predisposition, up to 20% of all prostate cancer patients reporting a positive family history and evidence of the genetic contribution to prostate cancer comes from the fact that the relative risk of prostate cancer increases markedly when the number of affected family members increases, or when the age at onset of the index case decreases. Nevertheless, the identification of highly penetrant genes responsible for the inherited predisposition to prostate cancer has been more difficult than for other types of common cancer, namely those of the breast and colon. We have collected a series of 462 probands with early-onset and/or familial/ hereditary prostate cancer and found a small proportion of the cases that are associated with germline mutations in known genes, namely HOXB13, BRCA2, and MSH2 [7, 8]. Ongoing studies using massive-parallel sequencing may allow identification of new genes associated with familial prostate cancer, which will be important to allow offering targeted screening to germline mutation carriers.

To characterise the intertumour heterogeneity regarding gene fusions in prostate cancer, tumour samples from a cohort of 200 patients with clinically localised PCa consecutively diagnosed and treated with radical prostatectomy were studied. The relative expression level of ETS family genes previously found rearranged in PCa (ERG, ETV1, ETV4, ETV5), Ewing sarcoma (FLI1, ERG, ETV1, ETV4, and FEV) or leukaemia (ERG and ETV6), and additional ETS genes found overexpressed (ELK4) or localised between two genes commonly rearranged (ETS2), was analysed with TaqMan low-density arrays, followed with FISH, reverse transcription polymerase chain reaction (RT-PCR), and sequencing analyses in selected cases [9]. This approach allowed the identification of rearrangements involving previously described ETS genes in 51.5%, 7%, 1%, and 0.5% for ERG, ETV1, ETV4, and ETV5 respectively. Additionally, this strategy allowed also the identification of a novel ETS gene involved in PCa gene fusions, namely FLI1, which was found to be fused with SLC45A3 [9]. Interestingly, we found four cases with two different ETS rearrangements in the index tumour, thus revealing intratumour genetic heterogeneity which most likely resulted from tumour collision of clonally independent neoplasias [9]. This work also unveiled a group of PCa with ETS rearrangements with unidentified 5’ fusion partners. This subgroup of patients was further studied by 5’ rapid amplification of cDNA ends and FISH and two novel 5’ fusion partners were identified (OR51E2 and UBTF), as well as two novel gene fusion combinations of previously described genes (SLC45A3-ETV4 and HERVK17-ETV4) [10]. Five previously described gene fusions involving ETV1 and ETV4 were also found. Moreover, to evaluate genetic markers of prognosis we studied a series of 200 diagnostic needle-biopsies with confirmed PCa with detailed clinicopathological data, including a ten-year disease-specific survival follow-up, in which the frequency of ERG rearrangements and relative 8q chromosomal gains were assessed by FISH. Whereas ERG rearrangement alone was not associated with clinical outcome, relative 8q gain predicted worse disease-specific survival in PCa patients both with and without ERG gene fusions, independently of Gleason score, clinical stage, and treatment modality [11].

Finally, we showed evidence of how the molecular heterogeneity of PCa might be explored therapeutically. We recently have shown that gastrin-releasing peptide receptor (GRPR) is an ERG and ETV1 target gene in prostate cancer, using a genome-wide scale and exonlevel expression microarray platform [12]. Our work showed that effective knockdown of GRPR in LNCaP and VCaP cells attenuates their aggressive oncogenic properties by decreasing proliferation, invasion, and anchorage-independent growth, while increasing apoptosis [13]. Using an antibody microarray we were able to validate known and identify new targets of GRPR pathway, namely AKT1, PKCε, TYK2, and MST1. We show that overexpression of these GRPR targets is restricted to prostate carcinomas harbouring ERG and/or ETV1 rearrangements [13], establishing their potential as therapeutic targets for these particular molecular subsets of the disease.

## Exosomes as biomarkers in cancer *Sonia A. Melo*

One of the most recently described mechanisms of intercellular communication is exosomes. Exosomes are extracellular membrane vesicles of a size range of 50 to 150 nm diameter, protected by a lipid bilayer. During their biogenesis, nucleic acids and proteins are encapsulated into exosomes. Additionally, we have recently demonstrated that exosomes are the carriers of circulating genomic DNA. Exosomes are released into the extracellular space and enter the circulation. All cell types described to date, such as immune cells, platelets or endothelial cells, release exosomes into the blood stream. Exosomes can transfer their cargo to various recipient cells. Recently, exosomes have emerged as putative biological mediators in cancer. This is attributed to the fact that cancer exosomes carry molecular signatures and effectors of the disease, such as mutant oncoproteins, oncogenic transcripts, non-coding RNAS (microRNAs, long non-coding RNAS, transfer RNAs, etc…) and double-stranded DNA. Intercellular trafficking of exosomes may contribute to horizontal cellular transformation, phenotypic reprograming, and functional re-education of recipient cells.

Exosomes derived from tumours carry molecular and genetic signatures associated with the cells or origin. Melanoma-derived exosomes contain the tumour-associated antigen Mart-1 and tyrosinase-related protein-2 (TYRP2). Exosomes from gastric cancer, breast cancer, and pancreas cancer carry members of the human epidermal growth factor receptor (HER) family. Therefore, we hypothesise that exosomes released by a tumour are heterogeneous and reflect the subpopulations of cancer cells co-existing in the tumour. Their heterogeneity is reflected in the different genetic material they carry that could affect other subpopulations of cells in the tumour and most likely regulate tumour progression and its ability to relapse after treatment by inducing plastic changes into therapy-resistant cells.

Routine screening of the general population for a cancer such as pancreatic adenocarcinoma using magnetic resonance imaging (MRI) or computed tomography (CT) would be prohibitively expensive and associated with a high false positive rate. We have recently demonstrated that glypican-1 protein on the surface of tumour-derived exosomes can be used as a biomarker for the early detection of pancreatic cancer. Using mass spectrometry analyses, we identify a cell surface proteoglycan, glypican-1 (GPC1), specifically enriched on cancer-cell-derived exosomes. GPC11 circulating exosomes (crExos) were monitored and isolated using flow cytometry from the serum of patients and mice with cancer. GPC1 crExos were detected in the serum of patients with pancreatic cancer with absolute specificity and sensitivity, distinguishing healthy subjects and patients with a benign pancreatic disease from patients with early- and late-stage pancreatic cancer. Levels of GPC1 crExos correlate with tumour burden and the survival of pre- and postsurgical patients. GPC1 crExos from patients and from mice with spontaneous pancreatic tumours carry specific KRAS mutations and reliably detect pancreatic intraepithelial lesions in mice despite negative signals by MRI. GPC1 crExos may serve as a potential non-invasive diagnostic and screening tool to detect early stages of pancreatic cancer to facilitate possible curative surgical therapy.

These results suggest the utility of exosomes in circulation as a detection and monitoring tool for pancreatic cancer, with an emphasis on its application in early detection, is a clinical challenge that remains to be addressed. Therefore, we propose to demonstrate that circulating exosomes-genetic content can be used as a promising non-invasive diagnostic and screening tool to detect early stages of pancreatic cancer that could aid the prospect of curative surgical therapy.

## Resolving tumour heterogeneity in solid FFPE samples by DEPArray digital sorting *Hans Peter Arnold*

Genomic profiling in solid tumour is notoriously challenging because of the presence of contaminating stromal and other kinds of cells. Microdissection is often attempted to enrich for the tumour cell fraction, but is inherently labour intensive and purity is limited especially when the tumour cells are highly intermingled with stromal cells, e.g. inflammatory cells. Also alternative approaches like the fluorescence-based cytometric analysis using fluorescence activated cell sorting (FACS) sorters to enrich tumour cells from disaggregated formalin fixed paraffin-embedded (FFPE) tumour tissues yield low efficiency and purity requiring a large input of cells material for instrument set-up and colour control. However the small sample size, e.g. biopsies, generally available in modern pathology impedes the use of conventional FACS for sorting. We present the results of genetic analysis for cell populations sorted by dieletrophoretic technology with our DEPArray system. This innovative technology solves two pressing problems in preparation of FFPE samples for genomic analysis: small sample size and unwanted admixture of stromal cells and other cell types. The presented procedure leads to robust and reproducible results, making it possible to unambiguously detect the true-positive variants and reliably analyse quantitative trait such as loss of heterozygosity (LoH) and copy number variations (CNVs), which cannot be evaluated precisely in unsorted FFPE samples. On several loci, we detected somatic mutations with 100% variant frequency in sorted tumour cell populations clearly indicating LoH and confirming 100% purity of sorted cells. Moreover, we identified and recovered cell subpopulations of potential epithelial-mesenchymal transition (EMT)-phenotype (positive staining for vimentin and pan keratine, DAPI staining yielded aneuploidy) exhibiting somatic mutations, different from tumour cells majority, and undetectable in DNA extracted from unsorted FFPE samples.

## Tissue microArray (TMA) technology: a tool to study tumour heterogeneity *Pasquale De Blasio*

To analyse tumour heterogeneity, the laser capture dissection (LCM) technology has been used in order to extract homogenous tumour cells or areas and apply antibody based studies. In a similar manner, the TMA can be used to extract tumour homogeneous areas from a tissue mass and use the selected areas or cores not only for antibody analysis but also for nucleic acids studies. The advantage in using TMAs is the absence of a laser and the speed in the molecular analysis since a large number of protein targets can be analysed simultaneously.

Over the years, the number of applications using the TMA technology has increased, it is being used not only in basic research studies but is slowly paving its way into diagnostics.

Various aspects of the TMA platform will be presented and its impact in Cancer Research and Diagnostics Applications. Tissue banks certainly have a central role in the preparation, storage, and distribution of tissue arrays (blocks and slides), and in the management of whole slide HD and TMA spots image.

Although several advances have been made to facilitate imaging and archiving of TMA specimens, automatic evaluation and quantitative analysis of TMAs still remains an important challenge for modern investigators.

Virtual pathology IT platform, will also be discussed, since it is a fundamental infrastructure in tissue banks since will prepare TMA’s for research, diagnostics, and educational purposes.

## Common aspects and diversities of tumour heterogeneity: is standardisation possible? The case of breast cancer *Anna Sapino*

It is well known that breast cancer is characterised by **intertumour** (different breast cancers among patients) and **intratumour** (multifaceted features within a given breast cancer) heterogeneity. Such heterogeneity can be appreciated at different levels, spanning from morphology to gene resolution. Although heterogeneity in breast cancer has been dissected by studies based on single cell analysis, morphology is often neglected. Morphological features define the histological type, which has been known to impact on breast cancer patient survival since the ‘90s [14]. Breast cancer can show mixed histotypes in about 25% of cases, featuring some characteristic special type areas. Some histological types such as tubular or cribriform carcinomas may be suitable for observations following surgery or may be treated by endocrine therapy alone, however St Gallen recommendations stress the fact that if another histotype is present the therapeutic approach should be remodeled according to the worse biological features or to the expression of markers of target of therapy [15].

Heterogeneity may represent a major issue in the neoadjuvant setting where core biopsies of large lesions are studied to plan the pre-surgery therapy. In this scenario multiple biopsies should be common practice in order to avoid improper sampling and to properly assess not only morphological but most importantly immunophenotypical features [16]. Among the four predictive markers routinely tested, oestrogen receptor (ER) is reported to be the least heterogeneous being expressed either at high or very low levels [17]. Conversely, progesterone receptor (PR) expression can be highly heterogeneous [18], leading to inter-observer variability, similar to ki-67 evaluation [19]. This marker heterogeneity suggests that multiple cores should be obtained for using tissue microarray procedure in routine [20].

HER-2 positivity is heterogeneous by definition, since we consider a 10% cut-off to assign samples to distinct score categories [8]. Score 0 and 1+ are more homogeneous in staining whereas score 2+ and score 3+ carcinomas show higher variability research excellence framework (REF). HER-2 heterogeneity is known to feature either two clearly distinct tumour clones or scattered HER-2 positive cells within a substantially negative tumour cell population [21]. Recently the mutational landscape of HER-2 heterogeneous carcinomas with two separate clones have been analysed and distinct driver genetic alterations in the different components have been demonstrated suggesting that HER-2 negative components are likely driven by genetic alterations not present in the HER-2+ components, including BRF2 and DSN1 amplification and HER-2 I767M somatic mutations [22].

On top of this **spatial heterogeneity, temporal heterogeneity** should also be taken into account, when comparing the immunoprofile of primary versus metastatic lesions: there is indeed evidence to demonstrate that tumours evolve over the course of the disease between the primary tumour and local or distant recurrences [23]. For instance CTCs frequently lack ER expression in metastatic breast cancer patients with ER-positive primary tumours and in general CTCs show a considerable intrapatient heterogeneity, which may reflect a mechanism to escape endocrine therapy [24]. Immunophenotype conversion in breast cancer is documented by several studies and poses important clinical questions, especially when the phenotype converts from positive to negative, which is the most frequent scenario, at least for hormone receptors. Some have reported that receptor conversion by immunohistochemistry in (non-bone) distant breast cancer metastases is relatively uncommon for ER and HER-2 and is more frequent for PR, especially in brain, liver, and gastrointestinal metastases [25].

Taken together the whole panorama of morphological and immunophenotypical features here described should be taken into account when high throughput molecular studies are performed to accurately assess the meaning and clinical impact of heterogeneity in breast cancer.

## Lung cancer heterogeneity *Lina Carvalho*

The WHO 2015 Lung Cancer Classification defines the main histological types of pulmonary carcinomas: adenocarcinomas, epidermoid carcinomas, neuroendocrine tumours, large cell carcinomas, adenosquamous carcinomas, sarcomatoid carcinomas, and pleomorphic carcinomas, and other rarer subtypes. Non-small cell lung cance (NSCLC) must include IHC profiling to highlight genetic alterations. Small biopsies can identify CK7/TTF1, CK5.6/p40, and vim/neuroendocrine markers.

Heterogeneity is often controlled by driver mutations present i.e. micropapillary (and cribiform/microglandular) pattern relates to metastatic potentiality and vimentin is usually positive in tumour cells; this antibody is also convenient to report sarcomatoid/pleomorphic carcinomas in biopsies.

Biopsies are the most reliable tissue to classify and treat pulmonary carcinomas because of the high percentage (70%) of tumours being diagnosed in advanced conditions.

Two repair/carcinogenic pools of adult stem cells have been recognised: the TRU –terminal respiratory unit concerning the respiratory bronchiole and adjacent alveolar duct/septae and the adult respiratory epithelium till the TRU.

Human induced pluripotent stem cells which have the potential to form a whole embryo have led to a better understanding of repair/ adaptation, and the possibilities of the intermingling of meso/ecto/endoderm in one single cell. After this preliminary study, CK7, CK5.7, TTF1, VIM, CD56, and Ki67 were shown to be sufficient for molecular pathology classification. Similar results have been obtained with chromium treatment of both fibroblasts and normal bronchial cells in contact where epithelial–mesenchymal transition was revealed.

Tobacco can cause cellular remodelling and adaptation of the basal cells in both TRU and respiratory epithelium, with consequent hyperplasia of vimentine positive cells, and TTF1 bronchial positive cells. This has been demonstrated in carcinomas arising after molecular transformation of less mature cells resulting in multi-patterned carcinomas and in pleomorphic carcinomas. This approach facilitated pulmonary carcinoma classification in biopsies but does not correlate directly with metastatic potential.

NGS demonstrated **the coexistence** of TP53/KRAS/CTNNB1/SMAD4/MET mutations with mutated EGFR. This may explain the variable response of the EGFR mutated tumours to the respective inhibitors. Prognosis will soon be explored by liquid biopsy sequencing in parallel with tumour re-biopsing to understand adaption to therapy. Primary lymph node metastasis studies revealed different morphology with the same molecular pathology where signet ring cell adenocarcinoma correlates with ALK fusion gene by FISH.

Co-existence of driver mutations has been demonstrated and NGS will allow more precise understanding of the clinical implications. When combining IHC and molecular pathology, the following cascades have been demonstrated in pulmonary carcinomas: epidermoid carcinoma–epidermal growth factor receptor (EGFR) and HER-2 polysomy, and CK7/vimentin for EMT non-pure epidermoid carcinomas; bronchial-pulmonary adenocarcinomas -non-smoking female—mut EGFR and excision repair cross complement group 1 (ERCC1) expression; micropapillary pattern with VIM/RB/ERCC1 expression; acinar/BA—lepidic/micropappilary patterns express TTF1, and mutated EGFR.

Higher ki67 and APC/ERCC1 expression correlates with stages IIA/IIIA while lower TTF1expression relates with mutated KRAS, mainly in papillary and solid patterns.

Endothelial marker expression, CD133, ALDH, VEGFR, PDGFR, and VASH are mainly associated with solid, acinar, and micropapillary patterns.

## Tumour heterogeneity in urothelial carcinoma *Arndt Hartmann*

Urothelial carcinoma of the bladder and upper urinary tract is a clinically, histopathologically and molecular heterogeneous disease. There are two types of urothelial carcinoma with distinct clinical and histopathological features. Papillary frequently noninvasive urothelial carcinomas are characterised by frequent recurrences and a low rate of progression to invasive and metastasising disease. This type of urothelial carcinoma shows only very infrequent genetic alterations which are limited to deletions of chromosome 9 and oncogenic driver mutations, like FGFR3 or PIK3Ca inducing proliferation. In contrast the second type of urothelial carcinomas is often characterised by primarily invasive disease, solid growth, poor differentiation, and a rapid progression to muscle invasive and metastasising disease. This type of urothelial carcinoma is developing through dysplasias and carcinoma *in situ* and is characterised by one of the highest load of genetic alterations and mutations of all tumour types. In this type of urothelial carcinomas different histopathological variants exist which are often intermixed and can be regarded as sign of the frequent stem cell character of these tumours. Examples for this are micropapillary, plasmocytoid, nested, or neuroendocrine differentiation. In the last year it has become evident that this type of high-grade urothelial carcinoma shows different intrinsic subtypes which reflect the hallmarks of breast cancer biology. These intrinsic molecular subtypes of urothelial carcinoma differentially express markers of urothelial differentiation.

Basal cell markers like basal cytokeratins and CD44 are detected in the basal type urothelial carcinoma and markers of the luminal umbrella cells like cytokeratin 20 or uroplakin are found in the luminal type of urothelial carcinoma. Interestingly, basal type urothelial carcinoma shows a decreased disease specific and overall survival (OS) in comparison to luminal type of urothelial carcinoma. Both intrinsic subtypes are associated with distinct genomic alterations like FGFR3and TSC1 mutations in the luminal and retinoblastoma (RB) alteration in the basal subtype. Furthermore luminal tumours express high levels of GATA3 and ER similar to luminal breast cancers and basal like tumours display enhanced MYC and E2F3 pathway signatures. The characteristic newly identified luminal and basal urothelial carcinoma subtypes were found in five different studies overall and showed in all studies a stable stratification by gene expression analysis. In a Consensus Meeting in Madrid in March 2015 IHC and RNA expression markers where defined which could be used in clinical studies in the near future. Most interestingly the different tumour types including a third p53-like subtype with an activated wild type p53 gene expression signature show a different response to neoadjuvant chemotherapy with best response in basal-like tumour and very poor response in p53-like tumours.

A second important and specific feature of urothelial carcinoma is a large degree of intrapatient heterogeneity. Urothelial carcinoma is frequently a panurothelial disease characterised by preneoplasias, multifocal tumours, and frequent recurrences. It is evident that the urothelium of patients with urothelial carcinoma is characterised by a field cancerisation with a multitude of molecular and histologically dysplastic urothelial changes. Many clonality studies could show that multifocal urothelial carcinomas are oligoclonal with a monoclonal disease because of intraluminal seeding and intraurothelial tumour spread in 80% of patients and the development of different tumour clones because of field cancerisation in 20% of patients. New whole genome sequencing studies identified genomic heterogeneity at a nucleotide and chromosome level in urothelial carcinoma. Two types of urothelial carcinoma with low and high genetic diversity and a high degree of mutational heterogeneity are found. Further studies should include assessment of histological normal but possibly genetically altered urothelial lessons to further define genetic heterogeneity in urothelial carcinomas. The high degree of genomic alterations could be used to develop immune therapies strategies.

## Session on clinical biobanks, how to tackle heterogeneity: heterogeneity in clinical research *Peter Riegman*

Biobanks are collections of biological samples and accompanying data which are provided for scientific research. Biobanks for medical research can contribute to heterogeneity in clinical research through introducing varieties in the quality of samples on different levels.

Medical research can be very demanding and often uses a variety of very sensitive high throughput tests. The high test sensitivity demands high quality samples and data, where ideally the samples are treated completely the same during the pre-analytical phase. This way, variations in sample quality are excluded and study bias is avoided. Biobank guidelines as well as certification and accreditation help to standardise the biobanks where the samples and data are stored safely and the procedures are completely standardised. Although there is a harmonising effect it leaves the individual biobanks free to choose the methods for collecting the samples.

Looking closer at the biobank procedures it is clear that not every step in the pre-analytical phase can be standardised. In case standardisation is not possible, avoidance of the procedure or avoidance by choosing a test insensitive to the introduced variation is recommended. The latter is very common for pathology departments, since FFPE samples have large quality variations, and pathology diagnostic tests are well known to be developed and become insensitive for these variations. If however avoidance is also not possible the variations need to be recorded. Tissues can be preserved in different ways resulting in different qualities and fit for purpose issues. The standard pathology archive stores FFPE tissues, which usually have poor DNA, protein, and RNA quality. On the other hand these samples have the best morphology for microscopic evaluation and are fit for purpose for many available techniques. However, the snap frozen tissues kept at ultra-low temperatures are seen as the golden standard.

A good biobank has standard operating procedures and a quality assurance and quality control programme in place and the best have these under an ISO certification/accreditation. Tissue banks are completely integrated in the Pathology department embedded in the health care pathways. In case the department becomes accredited the pathology tissue bank needs to take part because of this integration. This in combination with implementation of guidelines, best practices as well as dedicated and trained personnel helps to rule out bad samples when the study results are poor. This way, better support can be given to help obtain a good study result, which actually starts with good study design involving a multidisciplinary team where needed. Quality variety again becomes an issue when biobanks need to exchange samples in a multi-centre research project. In addition, the variations in patients and treatment contribute largely to these unwanted effects. Benchmarking of biobanks in proficiency testing (PT) and external quality assessmet (EQA) programmes could also help comparison of methods used within the biobanks and the hospitals to treat the patients. Furthermore the stimulation of multidisciplinary research teams for the study design and analysis can be key to tackle this problem.

Of course next to quality there are other important issues when managing a biobank. Total sample exchangeability also depends after quality issues on good access rules, taking care of the implementation of the proper legal, ethical and social issues, and data interconnectivity. Sustainability of a biobank is of major importance. Therefore it is important to realise there is an obligation towards the donors and stakeholders of the biobank to stay active and keep the samples available for a very long period. This does not mean a biobank only needs to store samples, ideally the samples need to be given out almost at the same rate as they come in. Best sustainability is reached when the biobank is financially covered by either an institutional budget or a cost recovery business plan. This can be a combination perhaps stimulated here and there with project money. Therefore a biobank must be efficient in its collection strategy. Last but not least, sustainability also knows a social factor where the biobank needs to take good care of its stakeholders (PI’s), donors, and the general public.

## Tumour heterogeneity studied at the protein level-Requirements for biobanking *Ulf Landegren*

The scope for molecular analyses in pathology and diagnostics is increasing rapidly: more classes of molecular targets are attracting interest, including epigenetic marks, exosomes, interacting proteins and rare mutant DNA in plasma. More and more examples of each of these categories are subject to study in research and later clinically, and these analyses are being performed on samples from more tissues and taken at more time points for each individual. This trend towards greatly increased molecular analyses has prompted the development of very high-throughput and low cost analytic techniques. The construction of suitable molecular tools for analysis of proteins and nucleic acids is a central aspect of work in my group, and in our spin-out company Olink.

We have developed proximity ligation assays for high-performance, high-throughput analyses of proteins in solution-phase patient samples, or *in situ* in pathological specimens. The assays use antibodies with attached oligonucleotides. Upon binding by a pair of such antibodies to a protein or a pair of interacting proteins the conjugated oligonucleotides can participate in ligation or extension reactions to give rise to amplifiable reporter DNA strands for highly sensitive and specific detection.

The *in situ* form of the assay, referred to as *in situ* PLA or Duolink®, uses localised amplification via a rolling circle mechanism of the reporter DNA strands that form, and it can offer enhanced specificity and sensitivity of protein detection in tissue sections, and analyses of patterns of protein interactions and modifications. Around 1000 users around the world are now using the methods for e.g. to investigate cellular signalling or to develop novel markers of diagnostic utility, and Ola Söderberg with colleagues have applied the technique to define cellular signalling cascades one by one or in low multiplex, but higher throughput assays are still under development.

Another variant of the assay, PEA or Proseek®, is used for detecting proteins in solution phase in samples such as plasma and other body fluids, and in lysates of tissues and even of single cells. This assay is used to measure sets of 92 proteins and four controls in 1 μl sample aliquots with excellent specificity and sensitivity. So far assays for some 800 proteins have been developed. In Uppsala alone, plasma samples from more than 40 000 individuals have been analysed for 3.5 million proteins in only just over one year by Dr Masood Kamali-Moghaddam.

Another local colleague, Ulf Gyllensten, used the point, evidence, analysis (PEA) method to measure the dependence of protein levels on genetic background as well as on age, sex, and several more factors. He demonstrated that it was possible to define more narrow normal ranges for many of the proteins, and thus more readily identify significant variation, using these sources of information. Another means to better evaluate protein levels is by investigating trends of increasing or decreasing protein levels in samples from the same individuals over time. In this regard we have found that sets of 96 proteins and controls can be conveniently measured from discs of paper punched out of dried blood spots that can be collected by the donors themselves, sent by mail, and stored at room temperature. This simple technique can form the basis for very inexpensive collections of consecutive samples from the same individuals, and provide a valuable means of identifying and validating protein markers for early detection of disease.

Caroline Gallant and Spyros Darmanis have demonstrated in yet unpublished work that the PEA technique permits measurements of sets of 90 or so proteins and similar numbers of transcripts in the same individual cells, to better measure cellular heterogeneity, e.g. in responses to putative therapeutic agents.

Finally, the ultimate level of measuring heterogeneity is at the level of single molecules. A newly developed super rolling circle amplication (RCA) technology allows individual detected DNA, RNA, or protein molecules to be finely distinguished in highly specific reactions and to be counted as digital objects for e.g. using regular flow cytometers, as developed by the two PhD students Lei Chen and Johan Björkesten. These techniques open the doors to highly precise quantitation, and they are suitable to identify rare DNA sequence variants in plasma that may reflect the presence of minimal residual malignant disease.

## Conclusion

This short meeting has posed some very important questions regarding the diagnosis and treatment of tumours which may seem initially to be the same but on further investigation reveal quite different characteristics. In the future it will be important to characterise better intertumoral and intratumoral differences to offer the most appropriate therapy.
